# Microbial community storm dynamics signal sources of “old” stream water

**DOI:** 10.1371/journal.pone.0306896

**Published:** 2024-09-24

**Authors:** Dawn R. URycki, Stephen P. Good, Byron C. Crump, Natalie C. Ceperley, J. Renée Brooks

**Affiliations:** 1 Water Resources Graduate Program, Oregon State University, Corvallis, OR, United States of America; 2 Department of Biological and Ecological Engineering, Oregon State University, Corvallis, OR, United States of America; 3 Department of Earth and Planetary Sciences, McGill University, Montréal, Canada; 4 College of Earth, Ocean and Atmospheric Sciences, Oregon State University, Corvallis, OR, United States of America; 5 Hydrology Group, Institute of Geography (GIUB) and Oeschger Center of Climate Change Research (OCCR), University of Bern, Bern, Switzerland; 6 Pacific Ecological Systems Division, Center for Public Health and Environmental Assessment, U.S. Environmental Protection Agency, Corvallis, OR, United States of America; City University of New York, UNITED STATES

## Abstract

Accurate characterization of the movement of water through catchments, particularly during precipitation event response, is critical for hydrological efforts such as contaminant transport modeling or prediction of extreme flows. Abiotic hydrogeochemical tracers are commonly used to track sources and ages of surface waters but provide limited details about transit pathways or the spatial dynamics of water storage and release. Alternatively, biotic material in streams is derived from thousands of taxa originating from a variety of environments within watersheds, including groundwater, sediment, and upslope terrestrial environments, and this material can be characterized with genetic sequencing and bioinformatics. We analyzed the stable water isotopes (δ^18^O and δ^2^H) and microbiome composition (16S rRNA gene amplicon sequencing) of the Marys River of western Oregon, USA during an early season storm to describe the processes, storage, and flowpaths that shape surface water hydrology. Stable water isotopes (δ^18^O and δ^2^H) typified an event response in which stream water is composed largely of ‘old’ water introduced to the catchment before the storm, a common though not well understood phenomenon. In contrast, microbial biodiversity spiked during the storm, consisting of early- and late-event communities clearly distinguishable from pre-event communities. We applied concentration-discharge (cQ) analysis to individual microbial taxa and found that most *Alphaproteobacteria* sequences were positively correlated (i.e., were mobilized) with discharge, whereas most sequences from phyla *Gammaproteobacteria* and *Bacteroidota* were negatively correlated with discharge (i.e., were diluted). Source predictions using the prokaryote habitat preference database ProkAtlas found that freshwater-associated microbes composed a smaller fraction of the microbial community during the stream rise and a larger fraction during the recession, while soil and biofilm-associated microbes increased during the storm and remained high during recession. This suggests that the “old” water discharged during the storm was likely stored and released from, or passed through, soil- and biofilm-rich environments, demonstrating that this approach adds new, biologically derived tracer information about the hydrologic pathways active during and after this event. Overall, this study demonstrates an approach for integrating information-rich DNA into water resource investigations, incorporating tools from both hydrology and microbiology to demonstrate that microbial DNA is useful not only as an indicator of biodiversity but also functions as an innovative hydrologic tracer.

## Introduction

Comprehensive understanding of watershed hydrology, particularly in the context of global environmental change, is relied on for accurate flood forecasts, management of stormflow and drinking water resources, and inferences about fate and transport of pollutants. However, key questions about stream and river water quality and quantity remain unanswered due to limits in understanding of the dynamics of catchment water transport flowpaths and associated biogeochemical processes [1, 2]. To investigate these areas, water resource scientists and engineers have deployed a number of geochemical techniques focused on abiotic solute and particulate concentrations (including stable isotopes) and their variability with stream discharge [m^3^s^-1^] to indirectly infer timing, sources, and chemical processing within catchments [3–5].

For example, naturally occurring geochemical tracers are commonly employed to assess streamflow response to precipitation events such as naturally occurring stable isotope ratios of water: ^2^H/^1^H (δ^2^H) and ^18^O/^16^O (δ^18^O). Because they describe the composition of molecules of water, these tracers are not subject to the same disadvantages of dissolved solutes or compounds, which may not be conserved during flow. Hydrograph separation is a common application of stable water isotope tracers and is typically based on a two-component mass balance approach [6] in which the fractions of streamflow composed of baseflow and event water are determined by comparing the isotopic ratios of water in the stream to ratios of the inputs, commonly pre-event streamflow (i.e., baseflow) and precipitation (though more advanced approaches consider additional inputs such as groundwater and soil water [7]). Stable isotope analysis has led to transformative conceptual insights in catchment hydrology, including the observation that pre-event “old” water, rather than “new” water introduced during precipitation events, dominates streamflow event response in many systems [7, 8]. However, the mechanisms that drive the predominantly old water stream response remain unclear [9].

Furthermore, isotopic signatures in some systems may be difficult to interpret. For example, recent work [10] demonstrated that isotopic variation between tributary streams in the Willamette Basin in Oregon, USA did not reflect the well-documented Rayleigh rainout effect, in which heavier molecules precipitate preferentially and precipitation subsequently becomes increasingly depleted with elevation and distance from the vapor source [11, 12]. Subsequent work in the same basin identified complex storage and release dynamics resulting from differences in lithology that channel water from outside the basin and explained why certain isotopic signatures were considerably more isotopically enriched than incoming precipitation [13]. Furthermore, in near surface [14] and deeper [15] groundwaters, isotopic signatures reflect the average, or at least a mix or intermediary value, of the annual precipitation, complicating efforts to apply isotopic analysis to trace subsurface water pathways. These complexities, including findings in the Marys River Basin of western Oregon, USA, where our study is located, underscore a broader need to develop new approaches to further develop our understanding of the dynamics of water storage and release to streams.

Accurately characterizing the movement of water through catchments requires identification of not only the origin of water, but also where it travels and is stored between the time it enters as precipitation and exits at a stream outlet. Transport pathways are a mix of surface and subsurface routes; thus solutes and other matter transported by water are governed by characteristics of the surfaces that water contacts along its path, including for example the available minerals. Examination of concentration-discharge (cQ) relationships of solutes is a widely applied analysis in geochemical tracer hydrology [16]. Typically the concentration of various tracers (e.g., major anions and cations such as calcium, magnesium, sodium, potassium, chloride, nitrate, and sulfate) are classified according to whether they decrease, increase, or remain stable during the streamflow rise and recession following a rainfall event; the behavior of different solutes (i.e., dilution, mobilization, or chemostasis, respectively) can reveal how water sources and flowpaths change during precipitation events, perhaps signaling differences in source geology or land use [17].

In contrast to the well-documented behavior of geochemical tracers, the dynamics and time scales of (micro)biological community changes associated with shifting water source environments and flowpaths are less established; yet advances in genetic profiling of aquatic DNA from alpine [18], karst [19] and volcanic [20] systems have demonstrated that microbial communities are hydrologically responsive indicators of subsurface pathways and processes. Importantly, because diverse microbial communities are well characterized with bioinformatics tools and databases (e.g, SILVA [21], ProkAtlas [22]), the presence and relative abundance of different microbial taxa may be linked to specific biological processes, living environments, and community dynamics. While environmental microbiologists have made advances in understanding their discipline through integration of hydrologic understanding [23, 24], the hydrologic community has made only limited advances in leveraging microbial knowledge to advance our field, despite clear evidence that microbial communities are highly sensitive to hydrology [25–28]. Genetic data have not been more widely adopted in hydrologic applications because of the complexity of genetic information, a limited understanding of microbiology among hydrologists, and a lack of methodological frameworks for combining hydrological and microbiological observations.

**Fig 1:**
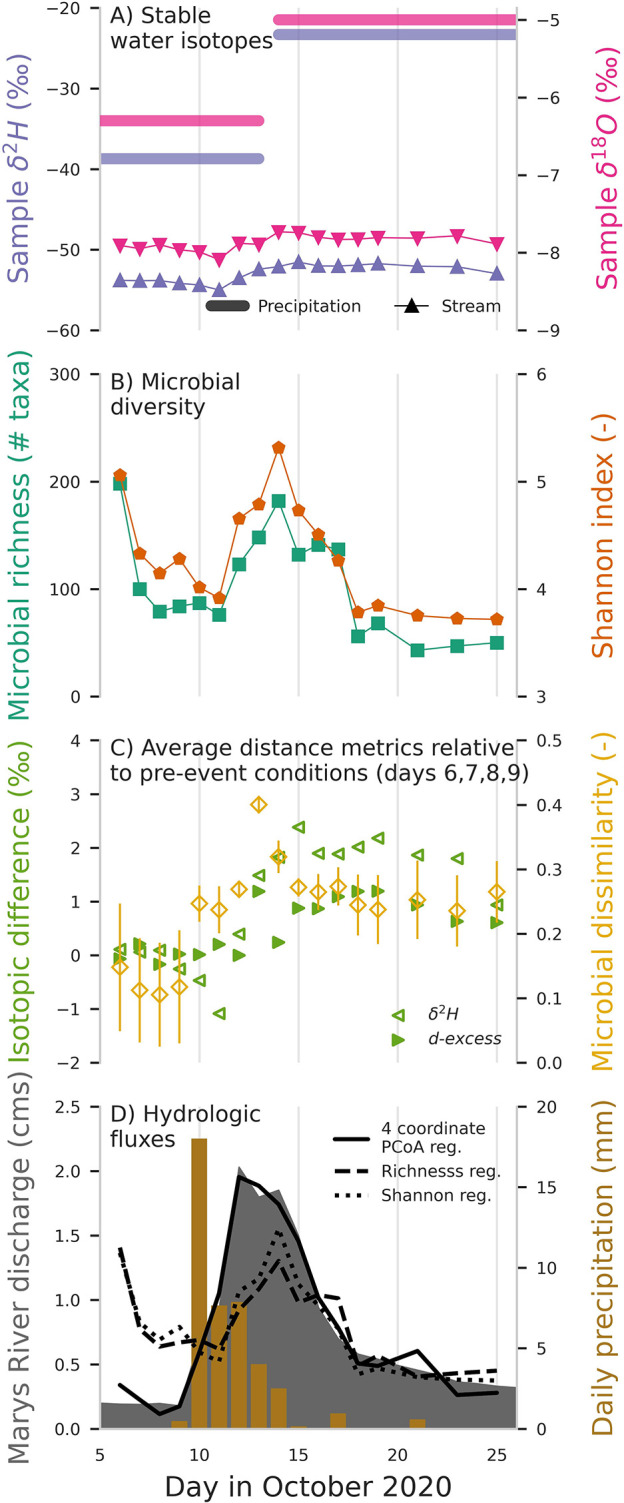
Microbial community diversity reflects the introduction of novel microbial taxa with the stream event response to an isolated precipitation event beginning 9 October 2020 on the Marys River, Oregon, USA. (**A**) Stable isotope ratios δ^2^H (purple) and δ^18^O (pink) measured in the stream (triangles) and in approximately 2-week aggregated precipitation (lines) demonstrate a subtle storm response. (**B**) Microbial community alpha diversity, including taxonomic richness (number of unique amplified sequence variants; teal squares) and Shannon index (red circles) exhibits dynamics similar to the storm hydrograph. (**C**) Mean difference from pre-event stable isotope ratios (Euclidean distance; green) and microbial community composition (Bray-Curtis dissimilarity; gold) illustrates the sensitivity of the microbial community to the event. Error bars indicate one standard deviation. Pre-event samples are 6–9 October. (**D**) Daily precipitation [mm] and Marys River daily observed (shaded) and modeled (lines) discharge [m3/s (CMS)]. Solid line is discharge predicted from multivariate linear regression of first four PCoA components (see Fig 2); dashed and dotted lines show discharge prediction from linear regression of taxonomic richness and Shannon index, respectively.

Here, we complement traditional hydrological observations with daily observations of the microbial community to interpret the stream response to a precipitation event. We first hypothesize that microbial community dynamics over the course of the storm will shift with changes in the hydrologic response of this system, and thereby reflect the source environments of microbes to the stream. Secondly, we hypothesize that these community and predicted source dynamics will show clear and significant shifts in microbial host environments, allowing us to better describe the complex spatial dynamics of storage, release, and transport of water through the catchment to the stream. We test these hypotheses in a typical mid-order watershed in the western United States by collecting daily DNA samples over the course of an isolated fall precipitation event on the Marys River in Oregon, USA, starting four days prior to the onset of precipitation and continuing for 16 days through the duration of the stream event response (Fig 1). We analyze and interpret the event response of conventional hydrologic tracers (δ^2^H and δ^18^O stable isotopes) as well as the dynamics of microbial community structure over this period. We then adapt a traditional hydrologic tracer analysis technique [16, 29] to characterize microbial taxonomic groups that are mobilized or diluted based on whether relative abundance is correlated or anticorrelated, respectively, with stream discharge. Finally, we leverage available knowledge of microbial ecology to make hydrologic inferences from patterns in taxonomic groups characterized as diluted or mobilized, as well as their likely source environments.

## Materials and methods

### Study area

Marys River is a small tributary of the Willamette River in Oregon, USA. The Marys River drains 394 km^2^ of steep (mean slope = 11.9°) forested (82.6%) area on the eastern slope (leeward side) of the Oregon Coast Range. The Marys River Basin is bounded by volcanic, sedimentary, and intrusive rock uplands [13, 30–32], underlain with basalt, sand- and siltstone, and some quaternary deposits [30, 32]. The Marys River Basin experiences a Mediterranean climate, with warm, dry summers and cool, wet winters. Most of the 1,830 mm of mean annual precipitation falls between November and June, with a rain-dominant regime below 400 m and a snow-dominant regime above 1,200 m; mean elevation is 290 m (range = 70–1, 300 m) [10, 33, 34]. The forests within the catchment are a mix of evergreen and deciduous, with limited cultivation and pasture (<20%). For further details on the watershed as well as topographic, geologic, and land cover maps of the catchment the reader is directed to [13].

### Datasets

We collected daily DNA samples at the Marys River stream gauge beginning three full days prior to a precipitation event (which began on the fourth day with a trivial amount of rainfall; Fig 1D) and continuing through the streamflow response and storm hydrograph recession; sample dates were 6–19 October, and 21, 23, and 25 October 2020 (Fig 1D). We collected all samples at approximately 0800 hours (range = 0730–0848 hours). To collect DNA samples, we obtained a water sample from the approximate center of the stream using a plastic bucket lowered from a bridge; we filtered and extracted DNA from streamwater as described in [35, 36]. A detailed description of equipment preparation and sampling methods is found in [37]. Water and DNA collections were conducted on a public waterway and in compliance with applicable laws; as such, explicit permission was not required. No protected species were sampled in this investigation.

After extracting and isolating the DNA, we PCR-amplified 16S rRNA genes with dual-barcoded primers targeting the V4 region (515f GTGCCAGCMGCCGCGGTAA, 806r GGACTACHVGGGTWTCTAAT; [38]) that were linked to barcodes and Illumina adapters following [39]. We sequenced 16S rRNA with Illumina MiSeq V.2 paired end 250bp sequencing. Sequences were processed with the QIIME2 bioinformatics pipeline [40] including quality control with DADA2 [41], and clustered into amplified sequence variants (ASVs). We classified sequence taxonomy with the SILVA 16S rRNA gene database v.132 [21] and removed ASVs classified as chloroplasts or mitochondria, or if they were not classified to the domains Bacteria and Archaea. The resultant dataset consisted of 248,286 sequences of 807 unique amplified sequence variants across the 17 stream samples (S1 Table). Each sample was composed of a median of 11,460 (range = 4,333–37,740) sequences. Alpha diversity, particularly richness, is inherently related to the number of sequences per sample, and rarefaction is a common technique to approximate even sampling depth, thus enabling meaningful comparison of alpha diversity across samples of unequal library size [42]. Therefore, we rarefied the dataset to 4,000 sequences per sample, resulting in a matrix of 4,000 × 17 = 68,000 counts of 620 unique ASVs. We analyzed these data as a matrix of the count of each unique ASV detected on each sample day and report these counts as relative abundance. We further grouped ASVs into the following eight categories based on taxonomy: classes 1) Actinobacteriota, 2) Bacteroidota, 3) Cyanobacteria, 4) Planctomycetota, 5) Verrucomicrobiota; orders 6) Gammaproteobacteria and 7) Alphaproteobacteria (both of Class Proteobacteria); and 8) Other (for ASVs that did not classify into any of the other seven groups). In microbiome research, individual ASVs are usually considered distinct taxonomic groups; therefore, we use the terms ASV and taxon interchangeably throughout this analysis.

For each water sample where DNA was collected, we collected a concurrent subsample of streamwater for stable isotope analysis by filling a 20 ml HDPE vial and wrapping it with flexible self-sealing film over the cap to prevent any evaporation. Samples were analyzed at the Stable Isotope Ratio Facility for Environmental Research (SIRFER) at the University of Utah to obtain the relative deviation from Vienna Standard Mean Ocean Water of stable isotope ratios deuterium (δ^2^H [‰]) and oxygen (δ ^18^O [‰]) following methods outlined in [43]. Accuracy and precision of streamwater samples is −0.43‰ and 0.16‰ for δ^2^H, and −0.06‰ and 0.03‰ for δ^18^O respectively, with accuracy and precision calculated as the mean and standard deviation of measurement errors on repeated measurements of secondary reference standards. We also obtained precipitation values of δ^2^H and δ^18^O for the study period from weekly bulk precipitation samples collected at the EPA Pacific Ecological Systems Division’s Corvallis campus, which is less than 6 km from our study site with a similar elevation, and within the Marys River Basin [13] and measured at EPA’s Integrated Stable Isotope Research Facility in Corvallis as part of a long-term precipitation isotope dataset for this location [10]. We used deuterium-excess (d-excess) to help separate shifts in source water during the event, and calculated it as *d-excess* = δ^2^H *×* δ^18^O [‰]. Deuterium-excess combines the information from both δ^*2*^H and δ^*18*^O and is sensitive to shifts in vapor source and evaporative processes [44]. We obtained streamflow records for the Marys River stream gage (#14171000) managed by the United States Geological Survey [45]. We aggregated sub-hourly data to daily mean discharge (ft^3^/s; converted to m^3^/s [CMS]) for the analysis period.

### Event flow responses

#### Microbial community diversity

Biodiversity is a characteristic measure of biological communities. We calculated alpha diversity, or the diversity within the community, for each sample day using two common metrics: taxonomic richness and Shannon’s index (Fig 1). Taxonomic richness is the total number of unique microbial taxa. Shannon index (*H*) is the entropy of taxa within a community, calculated as:

$$H=-\sum_{i=1}^{n}p_{i}{log}_{2}(p_{i}),$$

where *p*_i_ is the proportion of the total number of *n* ASVs represented by each unique ASV *i* [46]. (Although the base for the logarithm is arbitrary, when base 2 is used, the resultant quantity is described in units of bits for Shannon entropy; Shannon index is unitless, however.) Shannon index thus accounts for richness as well as evenness, or the relative abundance of each taxa. Larger values of *H* indicate greater diversity, and *H* is maximized when all taxa have the same relative abundance.

We then analyzed the extent to which Marys River post-event microbial communities differed from the pre-event communities. To quantify the difference between communities, termed beta diversity, we employed the Bray-Curtis dissimilarity metric [47], common in ecological studies. We calculated the Bray-Curtis dissimilarity between pairs of samples *u* and *v* as:

$$BC(u,v)=\frac{\sum_{i}\left|n_{i,u}-n_{i,v}\right|}{\sum_{i}\left|n_{i,u}+n_{i,v}\right|},$$

where *n*_*i*,*u*_ is the relative abundance of ASV *i* in sample *u*. Bray-Curtis dissimilarity ranges [0, 1], with unity indicating identical communities. We calculated the Bray-Curtis dissimilarity between pairs of samples (*u*_*l*_, *v*_*m*_), for all samples *l* = 6, 7, 8, …, 25 October and pre-event samples *m* = 6, 7, 8, 9 October. We analyze the difference between each sample community and the pre-event community as the mean Bray-Curtis distance for each sample from each of *s* = 4 pre-event samples as:

$$BC(u_{l})=\frac{\sum_{m}BC(u_{l},v_{m})}{s}.$$

To assess the microbial community response relative to a traditional hydrologic tracer, we compared microbial biodiversity and geochemical signature dynamics over the course of the stream event response. Analogous to BC dissimilarity, we calculated the mean distance between pre- and post-event deuterium ratios as the mean Euclidean distance (*d*) between each post-event sample and each of *s* = 4 pre-event samples as:

$$d(u_{l})=\frac{\Sigma_{m}d(\delta^{18}H_{l}-\delta^{18}H_{m})}{s}$$

We also calculated the mean distance between pre- and post-event *d*-excess as for deuterium ratios.

We visualized the relationships among microbial community samples with a principal coordinates analysis (PCoA) using the Python package *sci-kit bio* (v 0.5.6; http://scikit-bio.org; [48]). The PCoA maps similarity among samples, in this case Bray-Curtis dissimilarity, in multidimensional space. Samples mapped near each other are more similar than those mapped more distant. The relative position of each sample can be described by up to *n* coordinates for *n* samples, with each successive principal coordinate accounting for a diminishing proportion of the variation among samples. To assess how the differences between sample communities were associated with differences in stream discharge on the day of sampling, we fit an ordinary least squares regression between observed daily stream discharge and the first *p* principal components for *p* in the range [1, 17]. For comparison, we fit an ordinary least squares regression between stream discharge and δ^2^H and δ^18^O, and we also fit simple linear regressions between stream discharge and each of microbial richness and Shannon index. We evaluated model fit with the coefficient of determination (*r*^2^) between observed and modeled stream discharge for each model describing the percent of variation explained. We performed least square and linear regressions using *statsmodels* (v 0.12.2; https://www.statsmodels.org) and *scipy* (v 1.5.2; https://scipy.org/), respectively, both for the Python computing language.

#### Abundance–discharge relationships

We performed a regression analysis to characterize how individual microbial taxa respond to changes in discharge. Analogous to a cQ analysis, we assessed whether each microbial taxa exhibited a mobilization, dilution, or stasis response. To characterize taxa into these three groups, we performed simple linear regression between taxonomic relative abundance (ASV counts) and stream discharge for all taxa identified in at least three samples (i.e., days) across the entire study period 6–25 October. We classified each taxa as mobilized if the relationship between relative abundance and stream discharge exhibited a positive slope, diluted if the slope was negative, and static if the slope was not significant at a 90% confidence level. Uncharacterized taxa are those detected on too few (<3) days for linear regression.

To identify patterns in the response of taxonomic groups to hydrologic conditions, we quantified the fraction of sequences in each of the seven taxonomic groups that were classified as mobilized, static, or diluted in response to the precipitation event. To establish statistical confidence, we employed a shuffled surrogates method [49]. We first shuffled the relative abundance counts for each sample day to dissociate abundance counts from ASV IDs, then we shuffled the order of sample days to break patterns in stream discharge. In this way, we preserved the overall community diversity structure (i.e., relative abundance counts), but destroyed associations within taxonomic groups and with daily discharge. We determined the level of statistical significance of the observed fractions of mobilized, static, or diluted sequences in each taxonomic group by calculating the likelihood of observing similar fractions in 1,000 randomly shuffled surrogates. If the reported fractions of mobilized, static, or diluted sequences were above the 95^th^ percentile or below the 5^th^ percentile of the shuffled distribution, we rejected the null hypothesis that values are likely to be generated at random (p < 0.05). For example, if the 5^th^ and 95^th^ percentile of the fraction of *Gammaproteobacteria* identified as mobilized from 1,000 shuffled surrogates was 1% and 10%, respectively, we would reject the null hypothesis that values below 1% and above 10% are likely to be generated at random (*p* < 0.05).

#### Microbe source environments

We explored potential sources of microbial populations using the prokaryote habitat preference database ProkAtlas (http://prokatlas.bs.s.u-tokyo.ac.jp/; 2020-08-30 Updated pipelines; [22]) to identify sequence matches (97% identity) in its curated habitat-based database of 16S rRNA gene sequences. ProkAtlas output quantifies the proportion of habitats associated with relatives of each ASV. In our dataset, ASVs were associated with many habitats. To enable interpretation and comparison of these data, we first grouped similar habitats, reducing the number from 80 specific habitats down to eight environmental categories (hereafter environments, S2 Table). ASVs that were not assigned to a habitat were assigned to the “other” environmental category. We then converted the number of hits for each environment to a fraction of the total number of hits for all environments, such that for each ASV, the sum of the values for all environments is one (S2 Table). These values might thus be interpreted as the likelihood that a particular sequence originated from a specific source environment.

## Results

Event response in the isotope ratios (δ^2^H and δ^18^O) was subtle and gradual, and the small stable water isotope variation in the stream indicates that only a small amount of the substantial streamflow event volume was composed of recent precipitation. For example, the stream water δ^2^H isotope composition changed from −53.9‰ prior to the storm to a value of −52.1‰ on 13 October, after three days of precipitation (Fig 1A). Given that the precipitation was considerably more enriched (average δ^2^H = −38.7‰) than streamflow, a simple 2-component linear mixing model would suggest that >90% of the water on 13 October was water that was in the catchment prior to this storm while only ~10% of the water on this date was derived from this recent event. This modest increase in relative isotopic ratios in the stream despite the large influx to the watershed of highly enriched precipitation indicates that the bulk volume of event water in the stream was “old” water stored and then released from within the watershed, rather than “new” water introduced with the storm (S1 Fig). Discharge on the 13 October was 1.8 CMS, an increase of over 600% the pre-event discharge of 0.25 CMS. Thus, while the discharge increased 6-fold in response to this storm, only approximately 0.18 CMS of the flow on 13 October would be expected to be water that fell during this storm. A primarily “old” water stream event response is a common phenomenon [8], though isotope analysis offers little detail about the corresponding flowpaths or storage and release of this “old” water.

In contrast to the isotopic response observed in the two geochemical tracers, a dramatic influx of new and diverse microbial taxa was observable in the microbial community structure immediately following the onset of precipitation. Both alpha diversity curves, richness and Shannon index [46], broadly resembled the shape of the streamflow hydrograph (Fig 1B and 1D). Richness (the number of unique taxa) peaked with stream discharge from a pre-event mean of 80.7 (SD = 10.8) taxa to an event maximum of 178 taxa; Shannon index, which accounts for ecological richness as well as evenness through the relative distribution of taxa, followed a similar pattern, rising from a mean of 4.26 (SD = 0.12) to an event maximum of 5.26 (Fig 1B). The sharp, multidimensional event response of microbial diversity is characteristically different from the isotopic signature response (Fig 1C), reflecting the sensitivity of these tracers to different aspects of the hydrological response. The increase in microbial diversity indicates that new microbial taxa are being introduced to the stream, signaling a shift in the storage and release of water and (or) hydrologic pathways of the stream event flow.

**Fig 2:**
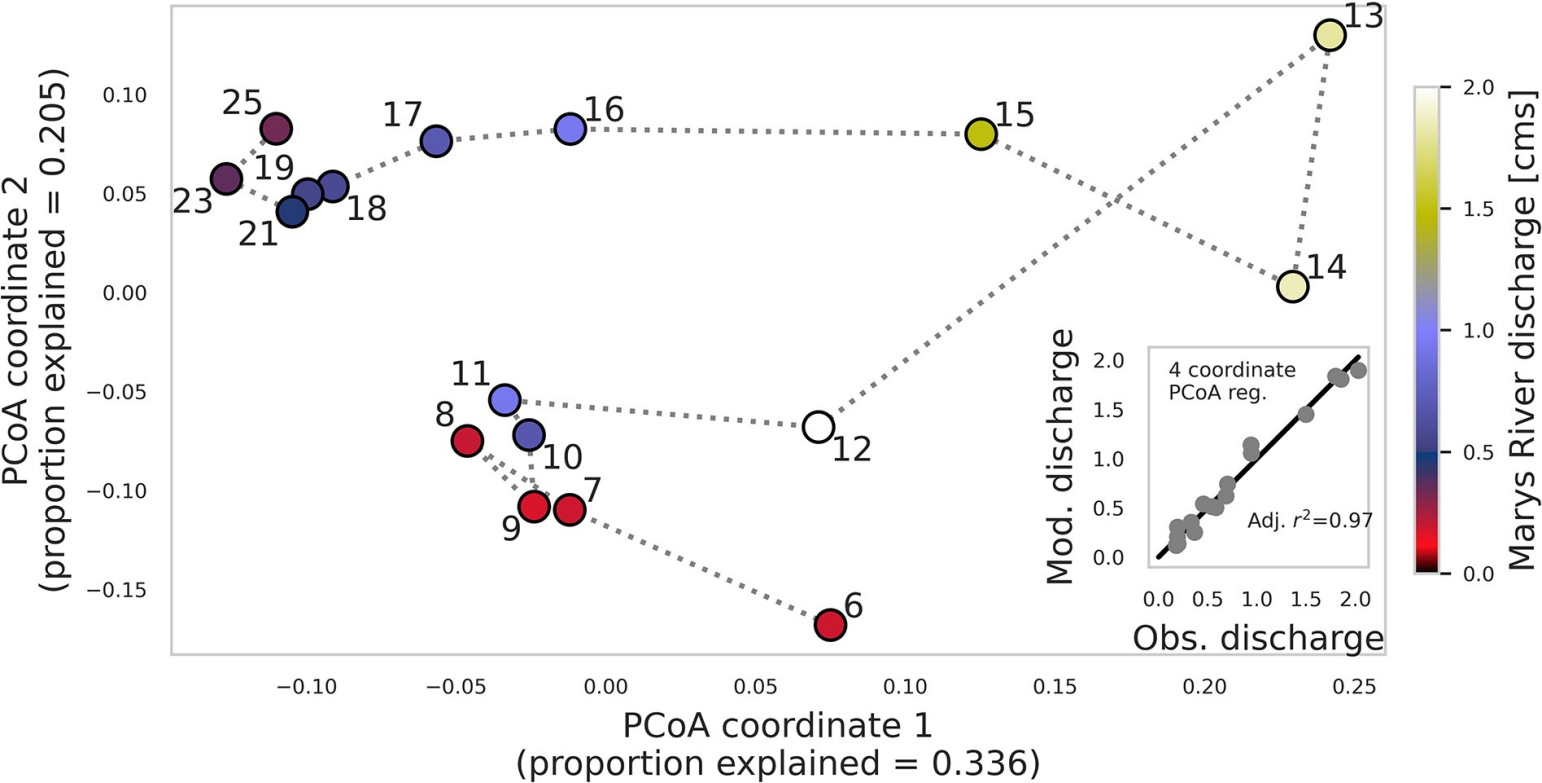
Principal coordinates analysis (PCoA) of streamwater microbial beta diversity illustrates a fundamental shift in microbial community structure that persists beyond the stream response to a precipitation event 6–25 October 2020 on the Marys River, Oregon, USA. Marker color corresponds to daily observed discharge [cms] on the date in October indicated by the marker number. Distances along axes indicate the proportion of differences in discharge explained by each principal coordinate; distances between points correspond to the magnitude of differences in microbial community structure, as measured by Bray-Curtis dissimilarity. Inset shows daily discharge modeled with multivariate linear regression of the first four principal coordinates versus observed discharge, with *r*^2^ adjusted for the number of predictors.

The precipitation event resulted in a transformation in community structure that was more complex than a one-dimensional increase in species diversity. Beta diversity, as measured by pairwise Bray-Curtis (BC) dissimilarity [47], was higher among pairs of pre- and post-event samples (BC dissimilarity = 0.27, SD = 0.04; Fig 1C) than among pairs of pre-event samples (BC dissimilarity = 0.13, SD = 0.02; Fig 1C), indicating that differences between pre- and post-event sample communities were greater than typical daily variability. Multidimensional visualization of microbial Bray-Curtis dissimilarity with principal coordinates analysis (PCoA) further illustrated a shift away from the pre-event community that was closely associated with baseflow stream discharge, with the most dissimilar community relative to pre-event communities occurring with peak discharge on 13 October (BC dissimilarity = 0.39, SD = 0.01; Figs 1C and 2). The shift in community structure persisted beyond the discharge response and the corresponding spike in alpha diversity, as observed by distinct pre- and post-event sample clusters in the PCoA (Fig 2), indicating that the event generated a microbial community that was not only more diverse, but also fundamentally different from the initial community.

This complexity of microbial response, as well as the tight relationship between community composition and discharge, is further evident through the regressions between microbial diversity and stream discharge. Simple linear regression between alpha diversity and stream discharge explained 45% (*p =* 0.07; Fig 1D) of the variation in stream discharge. Regression between alpha diversity and stream discharge modeled the general trends in the hydrograph but was unable to capture peak flow (Fig 1D). On the other hand, linear regression of just the first principal coordinate of BC dissimilarity was better (57% of variability explained, *p*<0.001). A multivariate linear regression of the first four principal coordinates of BC dissimilarity among microbial communities, captured both the trends and the peak flow exceptionally well, explaining 97% of the variation in stream discharge, with *r*^*2*^ adjusted for the number of predictors (Figs 1D and 2 [inset], Fig 1D and S2 Fig).

**Fig 3:**
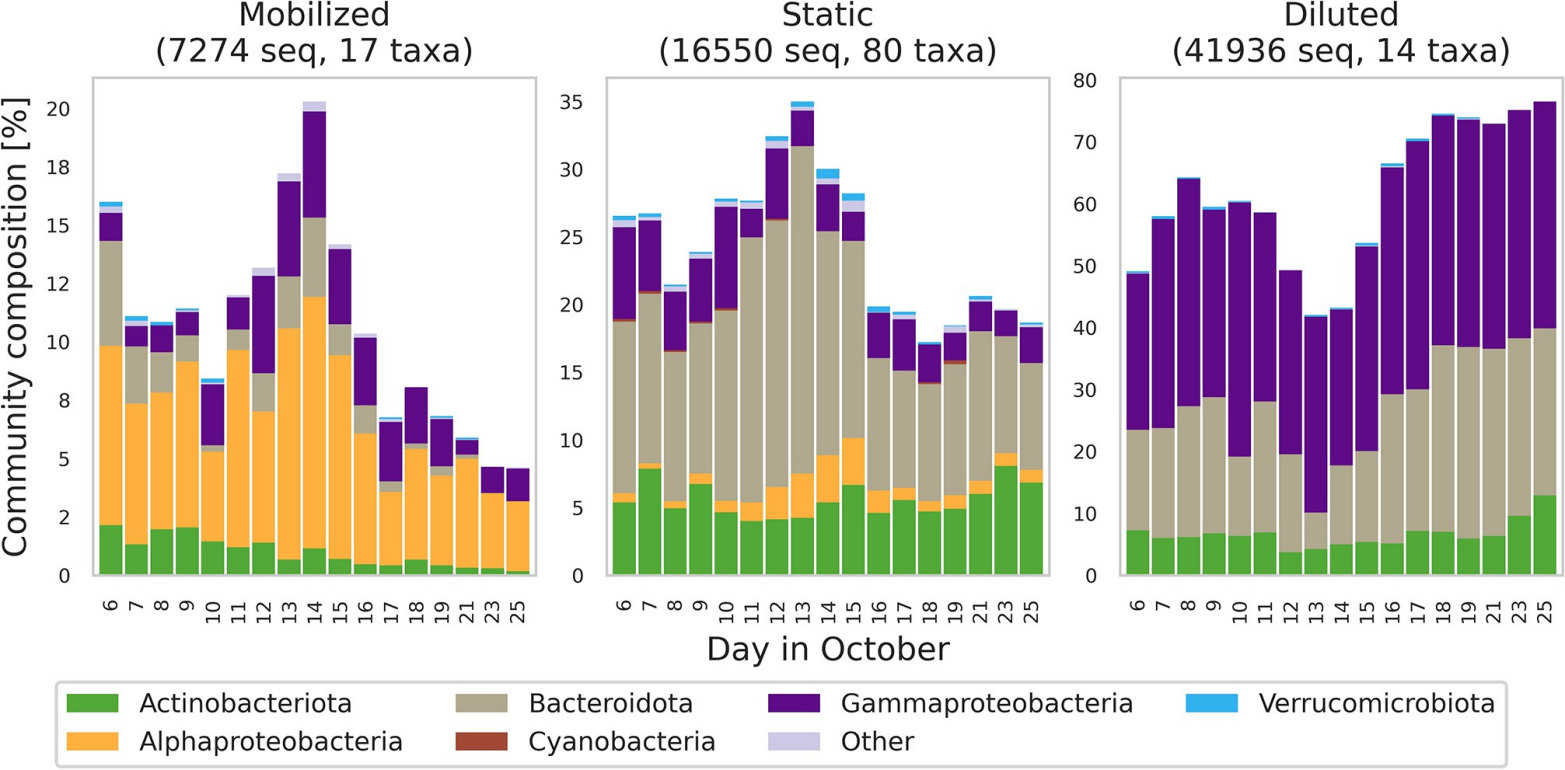
Increasing fractions of mobilized and static taxa in the microbial streamwater community, with a corresponding decrease in the fraction of diluted microbial taxa, detail the shift in microbial community structure associated with the stream response to a precipitation event beginning 9 October 2020 on the Marys River, Oregon, USA. Fraction is of the total abundance (number of sequences) identified in at least three samples and that were positively correlated (mobilized), not correlated (static), or negatively correlated (diluted) with stream discharge (*p* < 0.1).

We investigated some of the specific ways in which the community changed over the course of the storm by assessing the responses of different microbial taxa. We found that relative abundances of 17 microbial taxa exhibited significant positive correlations with streamflow volume over the course of the storm hydrograph (*p*<0.1), and we accordingly classified these taxa as *mobilized*, consistent with the nomenclature of commonly employed geochemical concentration-discharge analyses [16, 50]. Mobilized taxa consequently increased their representation in microbial community composition from a mean of 12.3% (SD = 2.4%) of the community in the four days before the storm to a peak of 20.3% of the community on 14 October, the day of peak discharge (Fig 3). On the other hand, we classified 14 taxa that were negatively correlated (*p<*0.1) with streamflow volume as *diluted*. Representation of diluted taxa in the microbial community decreased from a pre-storm mean of 57.7% to a low of 43.2% on 13 October (Fig 3). Community representation of 80 taxa that were not statistically correlated with discharge (i.e., *static* taxa, *p*>0.1), followed a pattern similar to that of mobilized taxa, generally increasing community representation on the rising limb of the hydrograph. Although static taxa lacked clear, statistically significant individual relationships with stream discharge, their collective representation in the microbial community may have increased during the hydrograph rise as a result of increasing alpha diversity with increasing event flow (Fig 1); increasing diversity reflects the introduction of a number of “new” taxa that were not likely detected in enough samples to identify significant relationships with discharge.

As flow receded, the fraction of the community mobilized (or static) with discharge likewise decreased, whereas diluted taxa increased. As with cQ analyses, these disparate responses to streamflow volume, and the subsequent change in community composition, signal a shift in contributions of microbes to the stream from distinct source environments within the catchment. In these analyses, we typically conclude that diluted species (chemical or microbial) originate from source environments that are either “turned off” or are overwhelmed by the inflow from different source environments, resulting in decreasing concentration of these species with increasing discharge. On the other hand, we often conclude that mobilized species originate from source environments that may be more readily tapped when discharge is higher, perhaps reflecting an increasing contributing area for event streamflow, in terms of catchment land area and(or) depth, for example. When we shuffled the relative abundance counts and sample order (see *Abundance–Discharge Relationships* above), these patterns vanished (S3 Fig), further supporting the inference that the patterns we observed are related to shifting sources of microbial community constituents with discharge dynamics.

**Fig 4:**
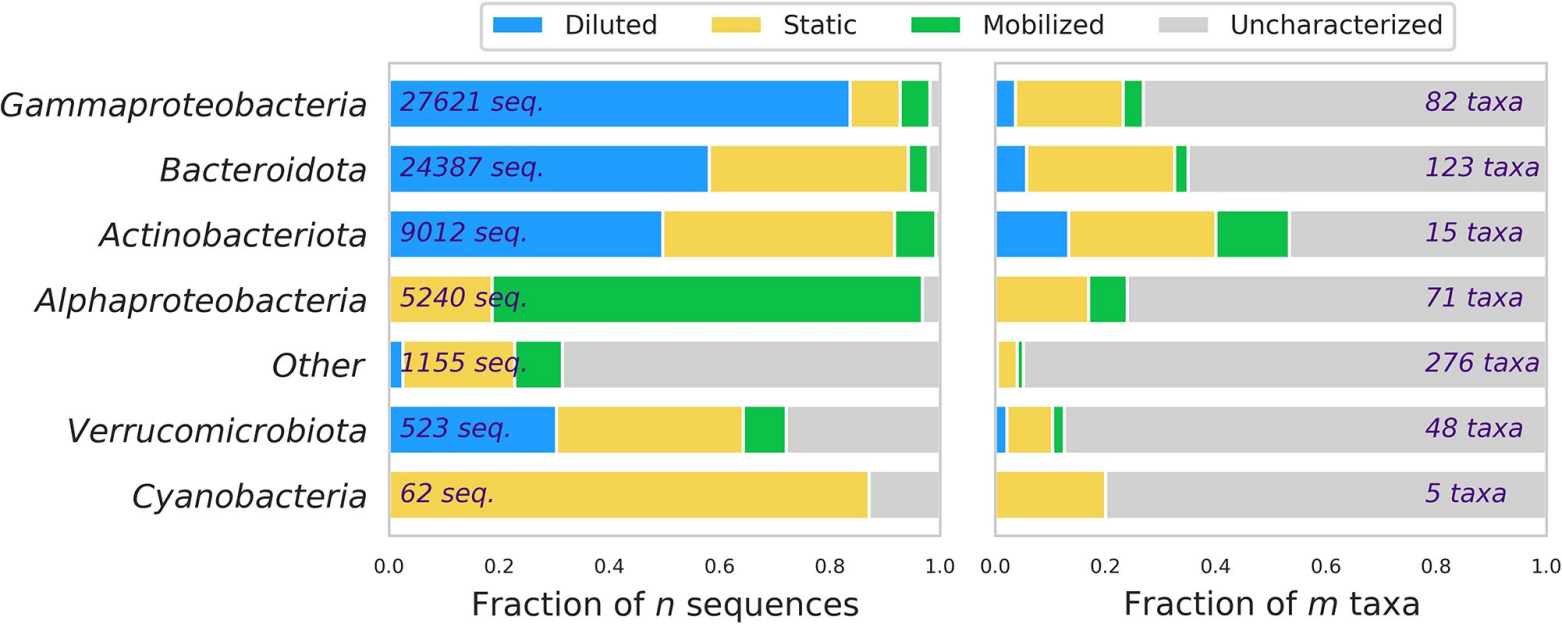
Patterns in the proportion of microbial taxonomic groups classified as mobilized, static, or diluted with streamflow volume offer evidence of shifting event flow sources in response to precipitation beginning 9 October 2020 on the Marys River, Oregon, USA. We collected 17 microbial community samples between 6–25 October. Proportions of microbial taxonomic groups identified as mobilized, static, and diluted are shown as a fraction of the total [relative] abundance of each amplified sequence variant (ASV) over the sampling period (left) and as a fraction of the number of unique taxa (right). Diluted, static, and mobilized ASVs are those that were identified in at least three samples and were negatively correlated, not correlated, or positively correlated with stream discharge (*p* < 0.1), respectively.

When we grouped microbes by higher taxonomic level (i.e., by Class), several groups exhibited strong patterns in abundance-discharge behavior. For example, 83.6% of sequences across taxa classified as Gammaproteobacteria were diluted with discharge, whereas only 5.3% were mobilized (9.0% were static; Fig 4). Similarly, 58.1% and 49.7% of sequences from Bacteroidota and Actinobacteriota were diluted, respectively, and only 3.6% and 7.5% were mobilized. In contrast, Alphaproteobacteria exhibited strong mobilization behavior, with 78% of sequences classified as mobilized and zero sequences identified as diluted. These patterns in discharge response among taxonomically similar groups suggests that microbial abundance-discharge behaviors are likely driven by common mechanisms interacting with ecological function, such as the release with a storm pulse from a terrestrial source microbial environment to the stream.

**Fig 5:**
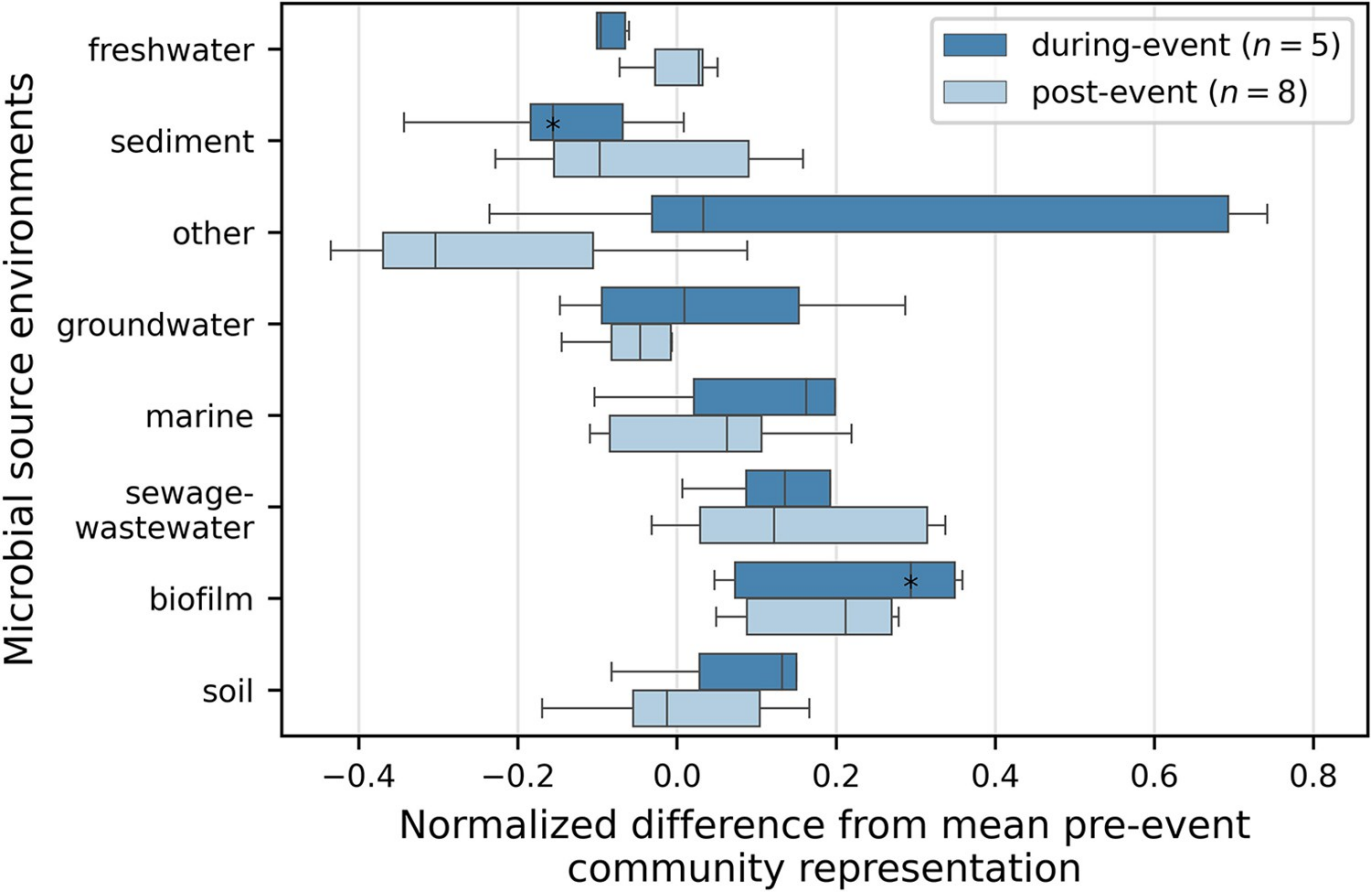
Difference in community representation (%) of each predicted source environment in the early event and post-event microbial communities, relative to the average pre-event community composition, illustrate shifting sources of microbes to the stream during an early season storm in October 2020 on the Marys River, Oregon, USA. Boxes and whiskers show the distribution of differences in representation from pre-event mean of each source environment in the sampled community during the early and post-event periods. A value of zero indicates no difference from the pre-event community. * indicates *p* < 0.1 (Mann-Whitney non-parametric *U* test).

To further explore the potential source environments of microbial populations, we used the ProkAtlas Online Toolkit [21] to predict habitat preferences for all taxa (97% identity). Results suggest diverse sources for Marys River microbes including freshwater, groundwater, soils, and sediments (S2 Table), that shifted rapidly during the storm, and changed more gradually during the flow recession (S4 Fig). The predicted source environments of individual ASVs identified as diluted and mobilized were highly variable (S5 Fig). However, across all taxa, the proportion associated with freshwater and sediment generally decreased during the storm, and for sediment-associated taxa, the decrease was statistically significant (Mann-Whitney non-parametric *U* test: *p* = 0.063). On the other hand, the proportion of taxa associated with marine environments, sewage–wastewater, and biofilm generally increased during the storm, and for biofilm-associated taxa the increase was significant (*p* = 0.063; Fig 5). These relationships were consistent when the same analysis was applied to the unrarefied dataset, and in fact were more statistically significant (decrease in freshwater: *p* = 0.063, in sediment: *p* = 0.016; increase in biofilm: *p* = 0.032; S10 Fig).

## Discussion

Microbial community diversity was highly sensitive to stream discharge and closely resembled the storm hydrograph, with the number of unique microbial taxa in the stream increasing more than twofold as stream discharge increased. The relationship between hydrology and microbial community composition, particularly diversity, is well-documented (e.g. [25]), although our study is the first we know of to observe this sensitivity to precipitation and hydrologic events at such a fine temporal (daily) resolution. For example, a strong positive relationship between microbial diversity and discharge was attributed to the greater influence of surface runoff following precipitation events in both a highly-impacted agriculturally-dominated system [26] and in an alpine spring [18]. Streamwater microbial communities, particularly in upper reaches, are known to develop primarily from terrestrial soil water inoculate [51], and it follows that storm runoff would thus introduce a surge of microbes. That the surge was composed of so many new taxa not previously detected in the pre-storm community reflects shifts in the flowpaths of water to the stream, possibly including an increasing contributing area, all of which fluctuate with variables such as antecedent soil moisture and event characteristics [52]. The sensitivity of the stream microbiome to precipitation events and the associated hydrologic response at a daily timescale thus offers promise for the utility of microbial communities as hydrologic source-water tracers.

The rich and dynamic response of the streamwater microbial community presents a characteristically different observation than the two- or three-dimensional (δ^2^H, δ^18^O, *d*-excess) isotopic response, offering hundreds or even thousands of data points to interpret. Even within the context of a spike in microbial diversity, for example, patterns were observable in the multidimensional community dynamics. As observed in the PCoA, microbial community composition exhibited a multivariate response more complex than the expression of event flow volume, reflecting a shift in the relative contributions from various reservoirs of surface and subsurface water (and microbes) and their shifting pathways to the stream. Furthermore, distinct pre- and post-event sample clusters in the PCoA indicate a transformation in the microbial community, which does not return to its pre-event structure as quickly as does streamflow volume. Similar to the way a soil water retention curve exhibits hysteresis (i.e., the wetting and drying processes are not symmetric; [53]), we observed a microbial community assembly dynamic in which the community composition following perturbation from the stream appeared to return to a composition resembling the initial pre-event composition, but following a more complex dynamic than a straightforward reversal of the perturbation (Fig 2). This asymmetric community perturbation and reassembly might be a result of the persistence of some taxa and the loss of other taxa from the community. The storage and release of soil water and microbes from various source environments to the stream depend on characteristics of a precipitation event as well as catchment attributes and antecedent conditions (i.e., [in]active flow paths or subsurface water storage reservoirs). The evolution of the microbial community before, during and after a storm event, both in terms of quality (which species) and quantity (species diversity) can thus be interpreted to intuit hydrologic processes. For example, if a certain microbial taxon has a clear association to a specific part of the catchment, its sudden appearance might indicate that area as a new (or activated) flow path. The stability or resilience (or lack of) in other aspects of the microbial community might indicate the persistent contribution of some flow paths or water storage reservoirs to the stream. The microbial dynamics we observed here thus support our hypothesis that these hydrologic data are encoded within microbial communities, expanding the scope and detail of hydrologic information detected within microbial communities in earlier work [37, 54].

That changes in microbial community composition result from shifting sources and pathways of water to the stream is further supported by the trends with discharge we identified in some of the phylogenetic groups. ProkAtlas-predicted source environments for Marys River bacteria were stable before the storm, and then shifted erratically during the rising limb of the hydrograph (starting on 10 Oct) resulting in increased soil, groundwater, biofilm, sewage-wastewater, and other sources (Fig 5 and S4 Fig). The proportion of these predicted source environments then decreased gradually during the falling limb of the hydrograph, demonstrating how microbial community composition may carry detailed information about water sources to rivers. Predicted source environments for individual ASVs identified as mobilized and diluted were less conclusive, possibly due to the limitations of current databases, which are still rapidly developing. Several abundant diluted ASVs were classified as members of typical planktonic freshwater bacterial genera such as *Limnohabitans*, *Rhodoluna*, and *Polynucleobacter* [55], suggesting that these riverine taxa were diluted during the storm by dispersal of bacteria from other environments. One diluted ASV related to the Bacteroidota genera *Pseudarcicella* was also found to be diluted by a pulse in flow event in the Murray River in Australia [56]. Predicted source environments for these taxa were diverse, but were dominated by freshwater. In contrast, abundant mobilized ASVs belong to Alphaproteobacteria and Gammaproteobacteria genera that have broad environmental distributions such as *Rhodoferax*, *Altererythrobacter*, and *Rhizobiales*, suggesting that their populations were enhanced by dispersal during the storm. However, ProkAtlas predictions of source environments for these ASVs were highly variable, and included many classified as freshwater, similar to the diluted ASVs.

Our ability to definitively characterize source environments of microbial taxa is therefore constrained both by existing available knowledge and tools, and also by the inherent limitations of generalizing about entire taxonomic groups. Taxonomic relationships are determined by shared evolutionary history, which does not universally imply functional similarity. For example, six out of eight *Flavobacterii* identified in our Marys River samples were diluted with discharge, in contrast to positive relationships between discharge and the relative abundance of some *Flavobacterii* identified in a karst alpine spring [19]. However, like many taxonomic groups, *Flavobacterii* exhibit tremendous diversity and have been associated with soil, freshwater, plants, animals, and humans [19, 55]. As research advances and our understanding of metabolism and ecology of individual taxa continues to develop, the capacity of the microbiome to contribute hydrologic information will also grow. Furthermore, it has been proposed that lower observed sensitivity of microbial community diversity to differences in land use and nutrient concentrations than to differences in temperature, hydrology, and organic matter may point to a high level of functional diversity within microbial cells, enabling them to function under a range of conditions [55]. Thus, meta-transcriptomics, in which sequences are characterized by functional genes rather than by taxonomy may offer additional opportunities to learn about hydrologic processes from the microbial community. Identification of key genes, for example those associated with nitrogen fixation or anaerobic respiration, may offer insight into the ecological functioning of diverse microbial assemblages, from which we have the potential to glean even more insight into ecohydrological function.

For this study, we opted to analyze the rarefied abundance matrix, for which we selected a random sample of 4,000 sequences from each library (i.e., sample day). It has been argued that conventional rarefaction methods result in an intolerable loss of data and should not be considered [57], particularly as rarefaction tends to result in fewer detections of rare taxa. In our case, however, our primary interest is in analyzing and reporting within-site alpha and beta diversity in the context of changes in streamflow during one precipitation event, with the objective of demonstrating the sensitivity of different aspects of microbial community composition to a stream event response, rather than to definitively characterize microbial biodiversity for comparison across sites or studies. Nevertheless, likely because our raw library sizes hardly spanned one order of magnitude (range = 4,333–37,740 reads), rarefaction only minimally reduced richness, from a median of 88.0 unique taxa per sample to 85.2 unique taxa per sample (mean of 500 rarefaction procedures) and resulted in no significant change to Shannon index (non-parametric Wilcoxon paired sample test mean *W* = 36.6, mean *p* = 0.146 for 500 procedures).

On the other hand, rarefaction did result in reduced detection of very rare taxa in our dataset. For example, after 500 iterations, the mean number of taxa lost to rarefaction was 153.8 (out of 807), which were detected 4.7 times on average. Of these rare taxa, 97.4% were detected on only one day out of 17 sample days (on 2 days: 2.5%, on 3 days: 0.6%, on 4 days: 0.7%; none were detected on more than 4 days). Given that abundance-discharge analysis relied on detection of significant relationships between relative abundance and stream discharge, elimination of these rare taxa reduced the noise in our dataset and strengthened our ability to derive relevant observations from these data. For example, the unrarefied data resulted in a poorer model fit in the regression between the 4-coordinate PCoA and discharge (S6 and S7 Figs), less distinction between the pre- and post-event stream microbiomes (though a during-event community is still distinguishable; S7 Fig), and fewer significant relationships between streamflow and microbial taxa (S8 and S9 Figs). Nevertheless, future analyses may use the full dataset or consider modified rarefaction approaches that seek to avoid the problem of “wasted data,” for example with repeated rarefaction [58–60], which could be useful for some analyses.

Future work may more closely identify and quantify the sources of microbial inputs to the stream. Microbial DNA collected from groundwater or soil from a range of depths and distances from the stream course could help develop a clearer picture of the sources, environments, and transport of microbial inputs to the stream. Models based on hydrologic principles and eDNA observations have been developed to upscale biodiversity to very high spatial resolution throughout a stream network [61]. Such a model could be adapted for microbial community analysis, enabling more detailed characterization of microbial community dynamics and, subsequently, characterization of stream event flow source dynamics at fine temporal resolution. Inputs from precipitation could also be considered [62].

## Conclusion

The hydrograph microbial community, as characterized by 16S rRNA amplicon sequencing, is a useful hydrologic dataset, encoding insights on hydrologic processes, such as sources and flowpaths of “old” water to the stream, which are not captured in more widely used tracers such as stable water isotopes. Our study validated previous observations of an “old”-water stream response in the Marys River, Oregon, USA, and furthermore, our analysis of the microbial response demonstrated the sensitivity of the microbial community, on a daily timescale, to a storm event and associated stream response. We adapt a common hydrologic tool, i.e., concentration-discharge analysis, to a microbial dataset to illustrate, for example, the activation of terrestrial sources and flow paths of water and microbes to the stream during the storm event. So, although isotope analysis is widely used to infer when, where, or how streamwater initially enters catchments as precipitation, microbial communities offer new information about the environments through which water travels as it moves through catchments to reach stream channels, as well as the dynamics of those contributing environments over the course of a storm (e.g., a reduction of freshwater-associated microbes and an increase in soil-associated microbes, as we observed). Continuing rapid developments in analytical methods and our understanding of microbial ecology will continue to improve the value and sharpen the detail of microbial communities as hydrologic datasets. This study represents a transdisciplinary effort, leveraging microbial sequencing and statistical tools from community ecology, as well as stable isotopes and solute analysis from hydrology, to advance the ongoing broad hydrological inquiry of what water is entering the stream, where it originated, and where it flowed on its way to the stream. With growing availability of new and more specific microbial datasets, such as meta-transcriptomics, many more such opportunities will become available.

## Supporting information

S1 Table Taxonomy of ASVs in the Marys River. Table also lists discharge response and unrarefied number of sequences. ASVs listed as diluted or mobilized are those for which relative abundance was significantly (p<0.1) related to streamflow volume.
(CSV)

S2 Table Predicted sources of individual ASVs identified as diluted and mobilized with stream discharge were highly variable, although many diluted ASVs were classified primarily as freshwater, and many mobilized taxa were linked to terrestrial environments during a major storm event beginning 9 October 2020 on the Marys River, Oregon, USA. Table lists discharge response, unrarefied number of sequences, and predicted source habitats and environmental categories of all ASVs. ASVs listed as diluted or mobilized are those for which relative abundance was significantly (p<0.1) related to streamflow volume. Undetermined taxa are those not statistically related to discharge or those detected in too few samples for linear regression. Source habitats were predicted with the ProkAtlas online toolkit, and ASVs with no predicted source habitats were assigned to the “other” environmental category.
(CSV)

S1 Fig Streamwater stable isotope ratios δ^2^H and δ^18^O measured in the stream (red ×) typify stream event flow dominated by “old” water, expressing a modest response to a major storm of highly enriched precipitation (~2-week aggregated precipitation ending 13 Oct [greed circle] and 2 Nov [blue diamond]) beginning 9 October 2020 on the Marys River, Oregon, USA. Streamwater data are for 17 stable isotope samples collected between 6–25 October. Long-term precipitation mean (gold), global meteoric water line (dotted), and local meteoric water line (solid) are shown.
(TIF)

S2 Fig Regression of the first four principal coordinates of microbial community beta diversity (Bray-Curtis dissimilarity) explains 97% of the variation in stream discharge in response to a major storm event beginning 9 October 2020 on the Marys River, Oregon, USA. Unadjusted *r*^*2*^ (light blue squares) and *r*^*2*^ adjusted for the number of predictors (dark blue circles) are similar. Simple linear regressions with alpha diversity (Shannon index [green circle] and taxonomic richness [green square]) explain more variation than stable water isotope ratios (δ^2^H and δ^18^O [purple circle]). Data are for 17 microbial DNA and water stable isotope samples collected between 6–25 October.
(TIF)

S3 Fig Shuffling sample order and relative abundance counts eliminates observed relationships between fractions of mobilized, diluted, and static microbial taxa with the stream discharge response to a precipitation event beginning 9 October 2020 on the Marys River, Oregon, USA. Fraction is of the total [relative] abundance (number of sequences) identified in at least three samples and that were positively correlated (mobilized), not correlated (static), or negatively correlated (diluted) with stream discharge (p < 0.1).
(TIF)

S4 Fig Predicted source environments for Marys River bacteria were stable and then shifted erratically during the rising limb of the hydrograph for a major storm event beginning 9 October 2020 on the Marys River, Oregon, USA. Increased soil, groundwater, biofilm, and unclassified sources then gradually declined during the falling limb of the hydrograph after 12 October 2020. Predicted environments were determined with ProkAtlas online habitat preference analysis tool with uncharacterized taxa included in the ‘other’ category.
(TIF)

S5 Fig Predicted source environments for bacterial taxa determined to be diluted or mobilized by a major storm event beginning 9 October 2020 on the Marys River, Oregon, USA. Each bar represents an individual taxa that was characterized as diluted or mobilized. Predicted environments were determined with ProkAtlas online habitat preference analysis tool.
(TIF)

S6 Fig Microbial community diversity (without rarefaction) reflects the introduction of microbial taxa with the stream event response to an isolated precipitation event beginning 9 October 2020 on the Marys River, Oregon, USA. (**A**) Stable isotope ratios δ^2^H (purple) and δ^18^O (pink) measured in the stream (triangles) and in approximately 2-week aggregated precipitation (lines) demonstrate a subtle storm response. (**B**) Microbial community alpha diversity (without rarefaction), including taxonomic richness (number of unique amplified sequence variants; teal squares) and Shannon index (red circles) exhibits dynamics similar to the storm hydrograph. (**C**) Mean difference from pre-event stable isotope ratios (Euclidean distance; gold) and microbial community composition without rarefaction (Bray-Curtis dissimilarity; green) illustrates the sensitivity of the microbial community to the event. Error bars indicate one standard deviation. Pre-event samples are 6–9 October. (**D**) Daily precipitation [mm] and Marys River daily observed (shaded) and modeled (lines) discharge [m3/s (CMS)]. Solid line is discharge predicted from multivariate linear regression of first four PCoA components (see Fig 2); dashed and dotted lines show discharge prediction from linear regression of taxonomic richness and Shannon index, respectively.
(TIF)

S7 Fig Principal coordinates analysis (PCoA) of streamwater microbial beta diversity without rarefaction illustrates a shift in microbial community structure during a precipitation event 6–25 October 2020 on the Marys River, Oregon, USA. Marker color corresponds to daily observed discharge [CMS] on the date in October indicated by the marker number. Distances along axes indicate the proportion of differences in discharge explained by each principal coordinate; distances between points correspond to the magnitude of differences in microbial community structure, as measured by Bray-Curtis dissimilarity. Inset shows daily discharge modeled with multivariate linear regression of the first four principal coordinates versus observed discharge, with *r*^2^ adjusted for the number of predictors.
(TIF)

S8 Fig Shifting fractions of mobilized, static, and diluted taxa in the microbial streamwater community without rarefaction in response to a precipitation event beginning 9 October 2020 on the Marys River, Oregon, USA. Fraction is of the total abundance (number of sequences) identified in at least three samples and that were positively correlated (mobilized), not correlated (static), or negatively correlated (diluted) with stream discharge (*p* < 0.1).
(TIF)

S9 Fig Patterns in the proportion of microbial taxonomic groups classified as mobilized, static, or diluted with streamflow volume (without rarefaction) offer evidence of shifting event flow sources in response to precipitation beginning 9 October 2020 on the Marys River, Oregon, USA. We collected 17 microbial community samples between 6–25 October. Proportions of microbial taxonomic groups identified as mobilized, static, and diluted are shown as a fraction of the total [relative] abundance of each amplified sequence variant (ASV) over the sampling period (left) and as a fraction of the number of unique taxa (right). Diluted, static, and mobilized ASVs are those that were identified in at least three samples and were negatively correlated, not correlated, or positively correlated with stream discharge (*p* < 0.1), respectively.
(TIF)

S10 Fig Difference in community representation (%) of each predicted source environment in the early event and post-event microbial communities (without rarefaction), relative to the average pre-event community composition, illustrate shifting sources of microbes to the stream during an early season storm in October 2020 on the Marys River, Oregon, USA. Boxes and whiskers show the distribution of differences in representation from pre-event mean of each source environment in the sampled community during the early and post-event periods. A value of zero indicates no difference from the pre-event community. * *p* < 0.1, ** *p* < 0.05 (Mann-Whitney non-parametric *U* test).
(TIF)
