# Supplementary figures and images for: Microbial community storm dynamics signal sources of “old” stream water
Source: PLoS One. 2024 Sep 24;19(9):e0306896. doi: 10.1371/journal.pone.0306896 (PMC11421800; doi:10.1371/journal.pone.0306896)

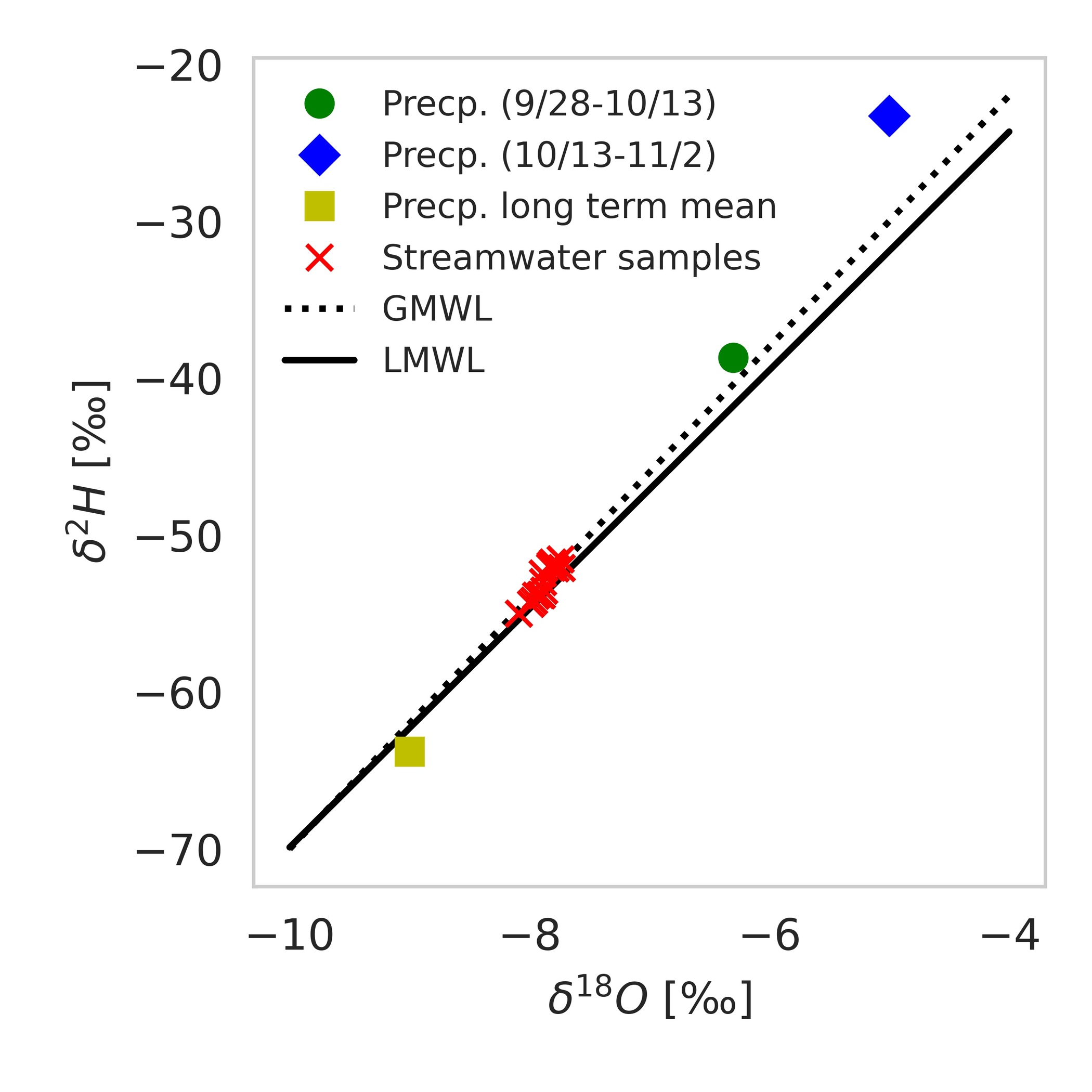

Supplement: S1 Fig — Streamwater data are for 17 stable isotope samples collected between 6–25 October. Long-term precipitation mean (gold), global meteoric water line (dotted), and local meteoric water line (solid) are shown. (TIF) [file pone.0306896.s003.tif]

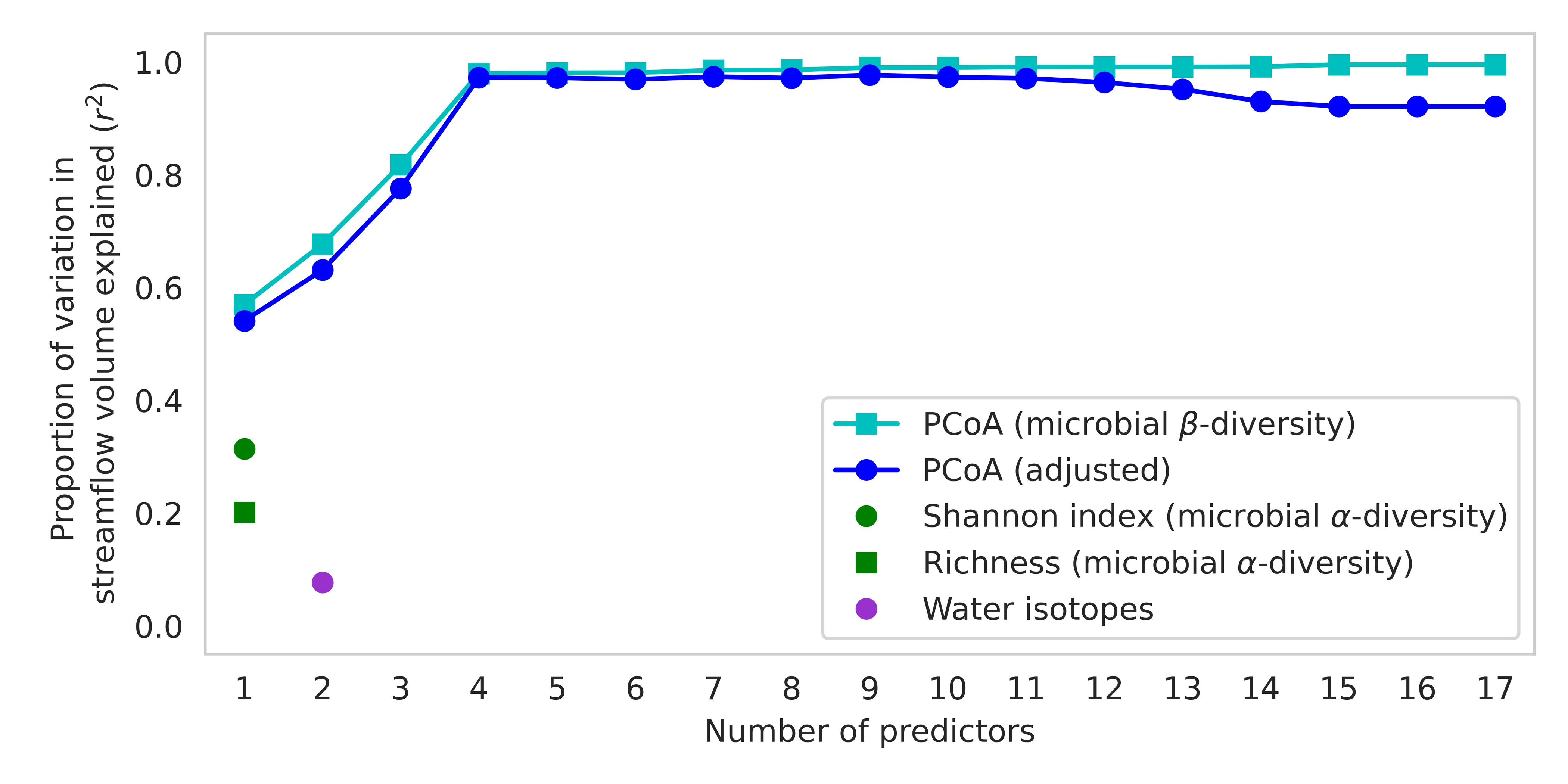

Supplement: S2 Fig — Unadjusted r2 (light blue squares) and r2 adjusted for the number of predictors (dark blue circles) are similar. Simple linear regressions with alpha diversity (Shannon index [green circle] and taxonomic richness [green square]) explain more variation than stable water isotope ratios (δ2H and δ18O [purple circle]). Data are for 17 microbial DNA and water stable isotope samples collected between 6–25 October. (TIF) [file pone.0306896.s004.tif]

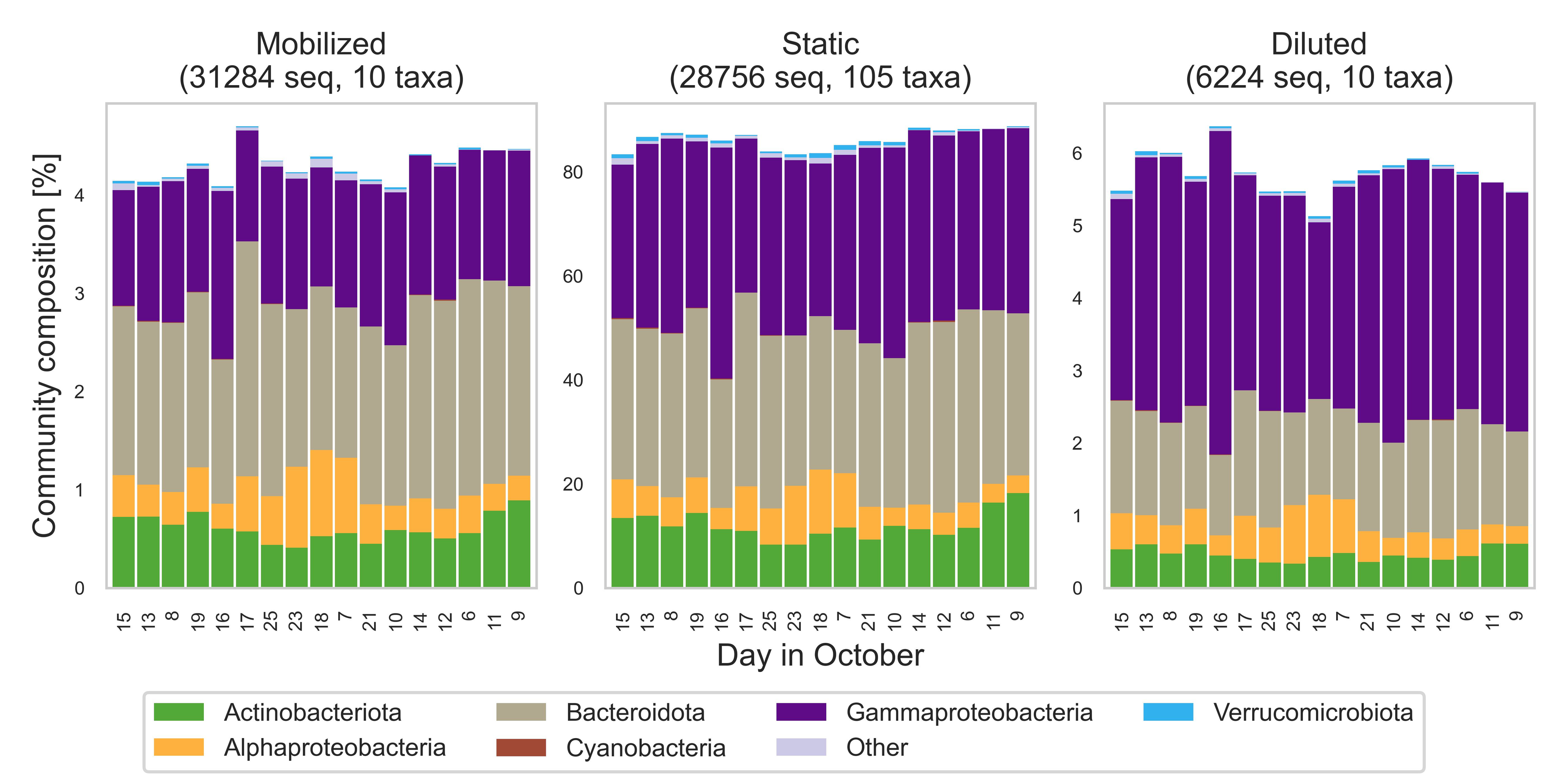

Supplement: S3 Fig — Fraction is of the total [relative] abundance (number of sequences) identified in at least three samples and that were positively correlated (mobilized), not correlated (static), or negatively correlated (diluted) with stream discharge (p < 0.1). (TIF) [file pone.0306896.s005.tif]

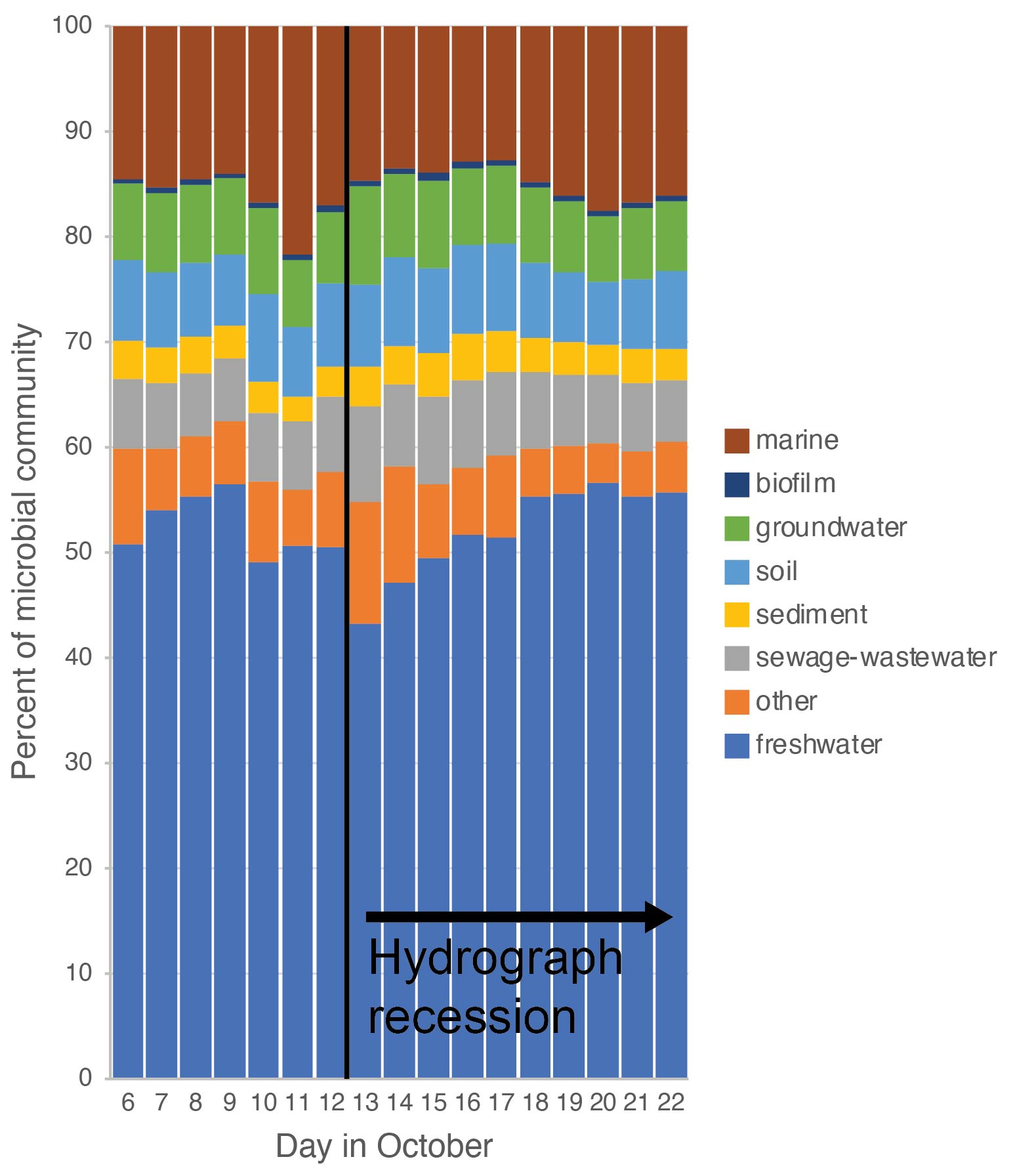

Supplement: S4 Fig — Increased soil, groundwater, biofilm, and unclassified sources then gradually declined during the falling limb of the hydrograph after 12 October 2020. Predicted environments were determined with ProkAtlas online habitat preference analysis tool with uncharacterized taxa included in the ‘other’ category. (TIF) [file pone.0306896.s006.tif]

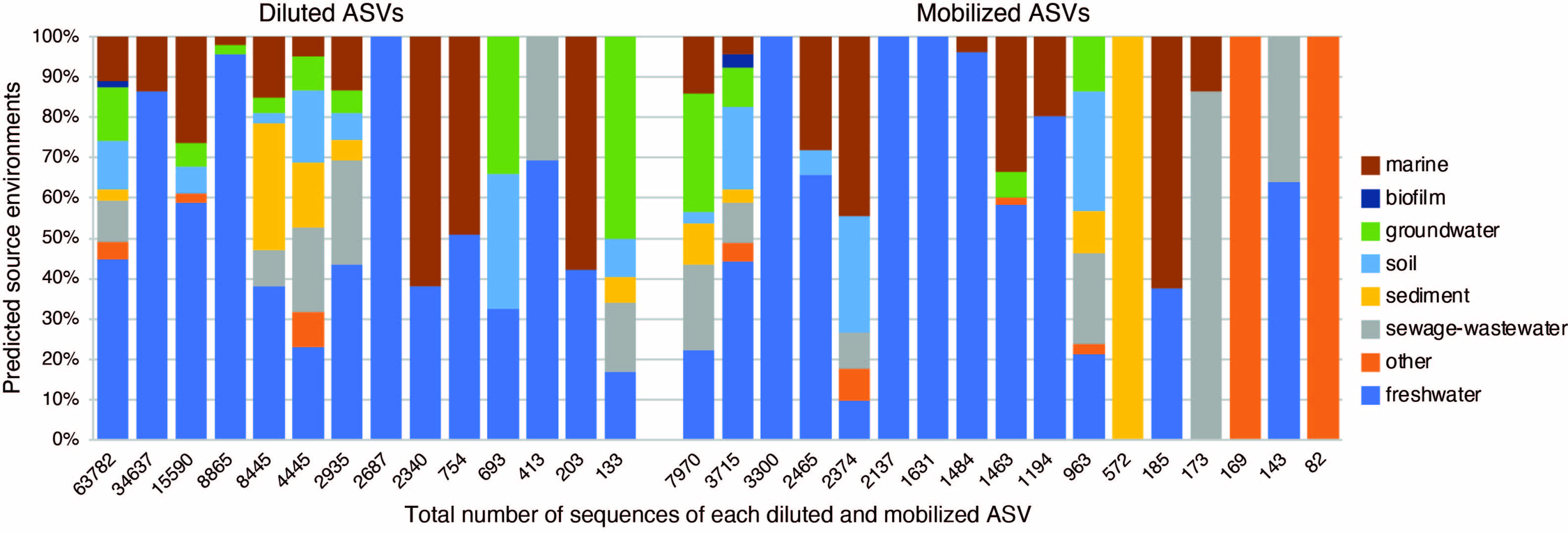

Supplement: S5 Fig — Each bar represents an individual taxa that was characterized as diluted or mobilized. Predicted environments were determined with ProkAtlas online habitat preference analysis tool. (TIF) [file pone.0306896.s007.tif]

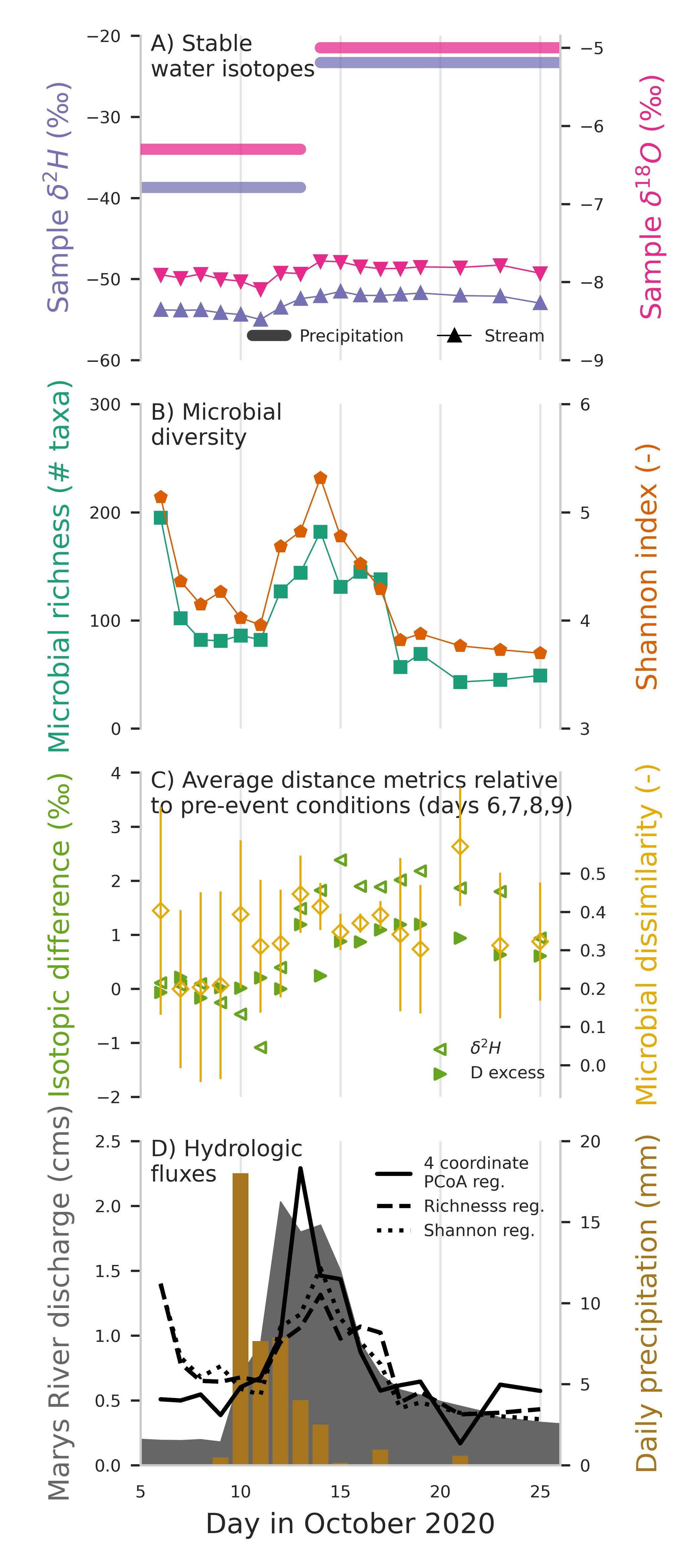

Supplement: S6 Fig — (A) Stable isotope ratios δ2H (purple) and δ18O (pink) measured in the stream (triangles) and in approximately 2-week aggregated precipitation (lines) demonstrate a subtle storm response. (B) Microbial community alpha diversity (without rarefaction), including taxonomic richness (number of unique amplified sequence variants; teal squares) and Shannon index (red circles) exhibits dynamics similar to the storm hydrograph. (C) Mean difference from pre-event stable isotope ratios (Euclidean distance; gold) and microbial community composition without rarefaction (Bray-Curtis dissimilarity; green) illustrates the sensitivity of the microbial community to the event. Error bars indicate one standard deviation. Pre-event samples are 6–9 October. (D) Daily precipitation [mm] and Marys River daily observed (shaded) and modeled (lines) discharge [m3/s (CMS)]. Solid line is discharge predicted from multivariate linear regression of first four PCoA components (see Fig 2); dashed and dotted lines show discharge prediction from linear regression of taxonomic richness and Shannon index, respectively. (TIF) [file pone.0306896.s008.tif]

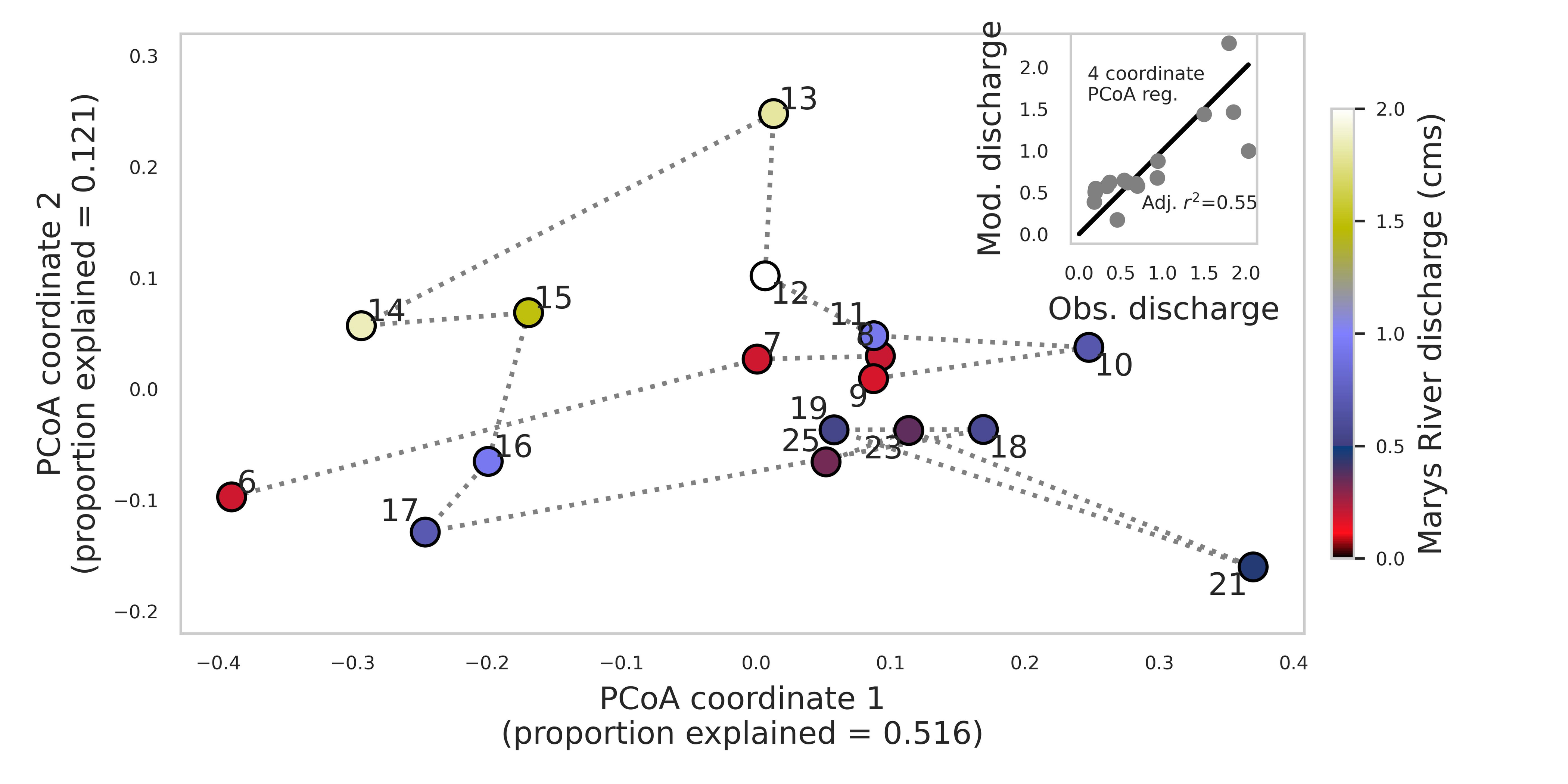

Supplement: S7 Fig — Marker color corresponds to daily observed discharge [CMS] on the date in October indicated by the marker number. Distances along axes indicate the proportion of differences in discharge explained by each principal coordinate; distances between points correspond to the magnitude of differences in microbial community structure, as measured by Bray-Curtis dissimilarity. Inset shows daily discharge modeled with multivariate linear regression of the first four principal coordinates versus observed discharge, with r2 adjusted for the number of predictors. (TIF) [file pone.0306896.s009.tif]

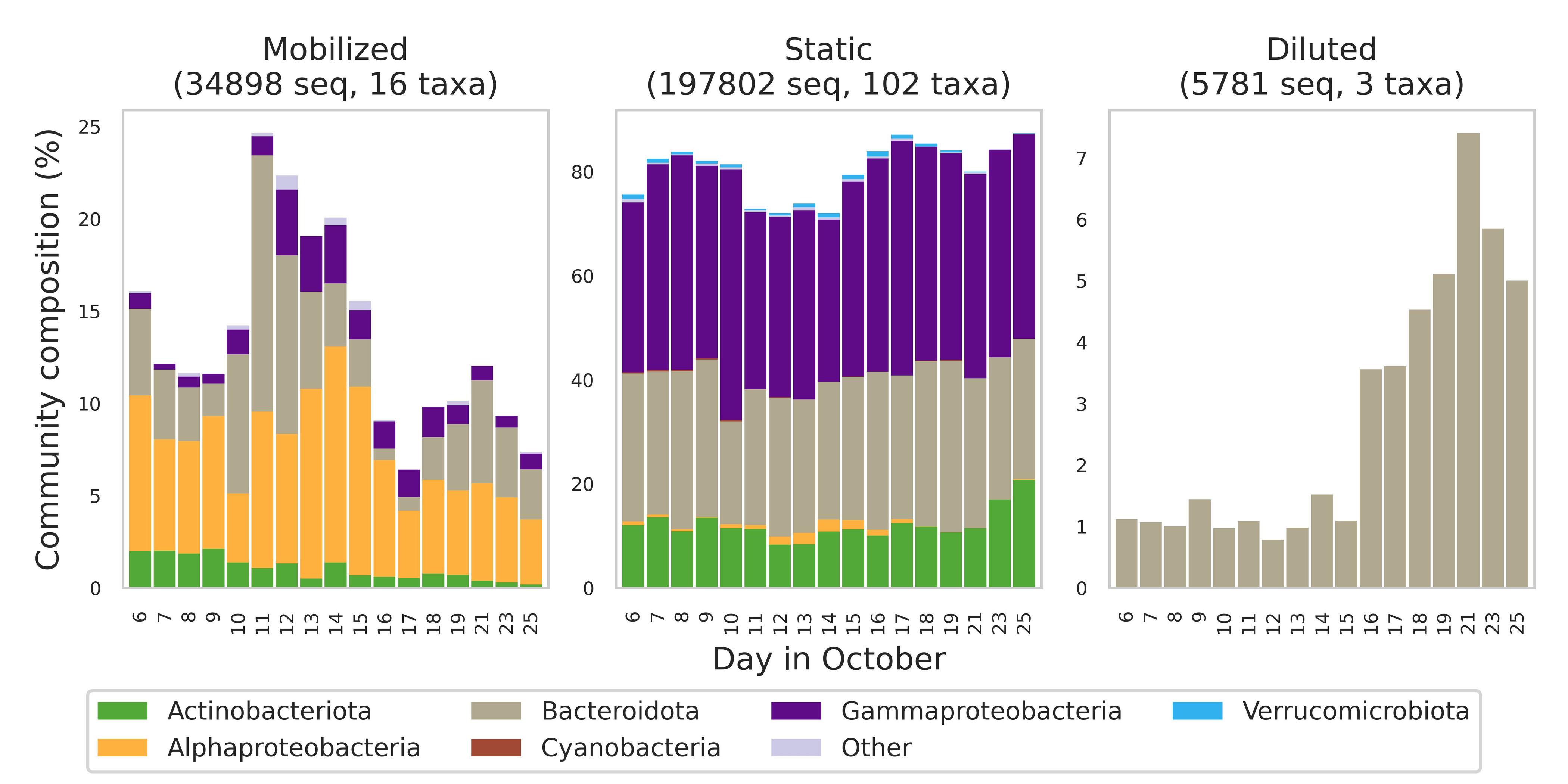

Supplement: S8 Fig — Fraction is of the total abundance (number of sequences) identified in at least three samples and that were positively correlated (mobilized), not correlated (static), or negatively correlated (diluted) with stream discharge (p < 0.1). (TIF) [file pone.0306896.s010.tif]

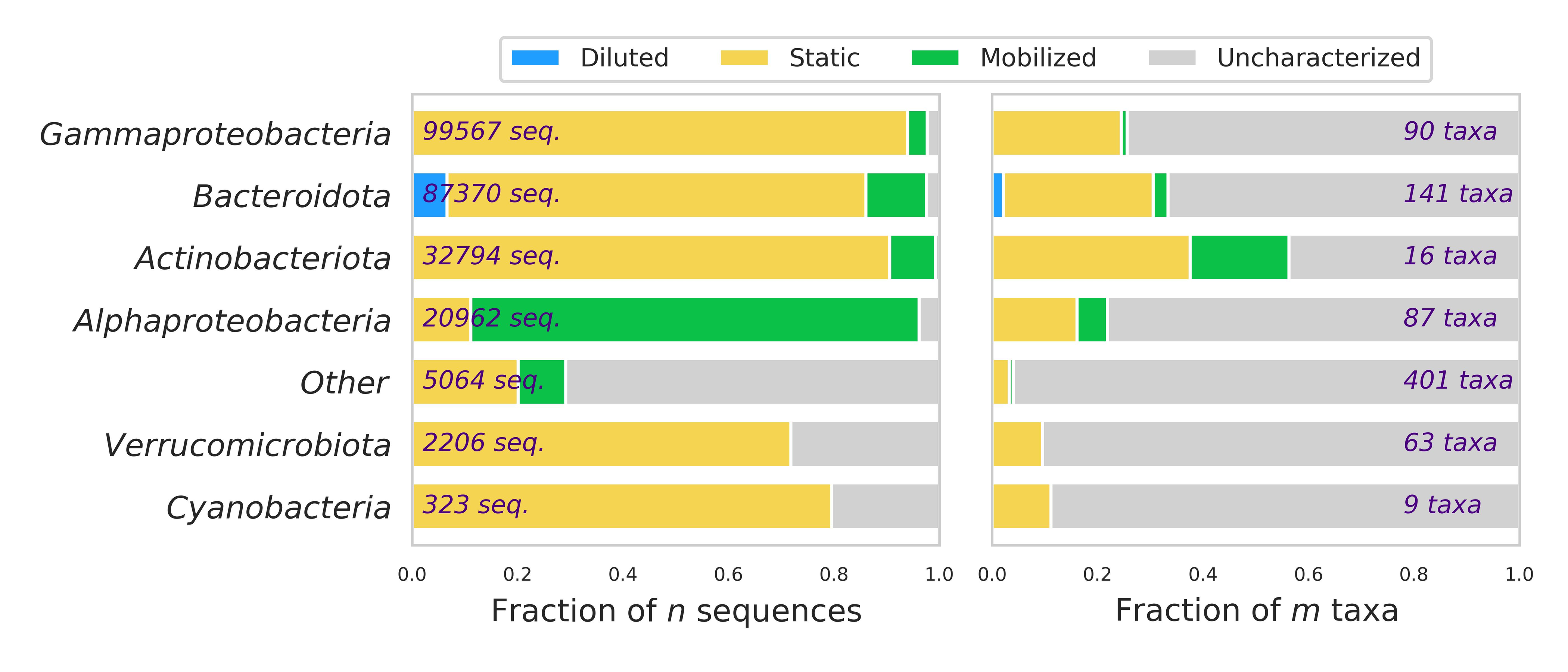

Supplement: S9 Fig — We collected 17 microbial community samples between 6–25 October. Proportions of microbial taxonomic groups identified as mobilized, static, and diluted are shown as a fraction of the total [relative] abundance of each amplified sequence variant (ASV) over the sampling period (left) and as a fraction of the number of unique taxa (right). Diluted, static, and mobilized ASVs are those that were identified in at least three samples and were negatively correlated, not correlated, or positively correlated with stream discharge (p < 0.1), respectively. (TIF) [file pone.0306896.s011.tif]

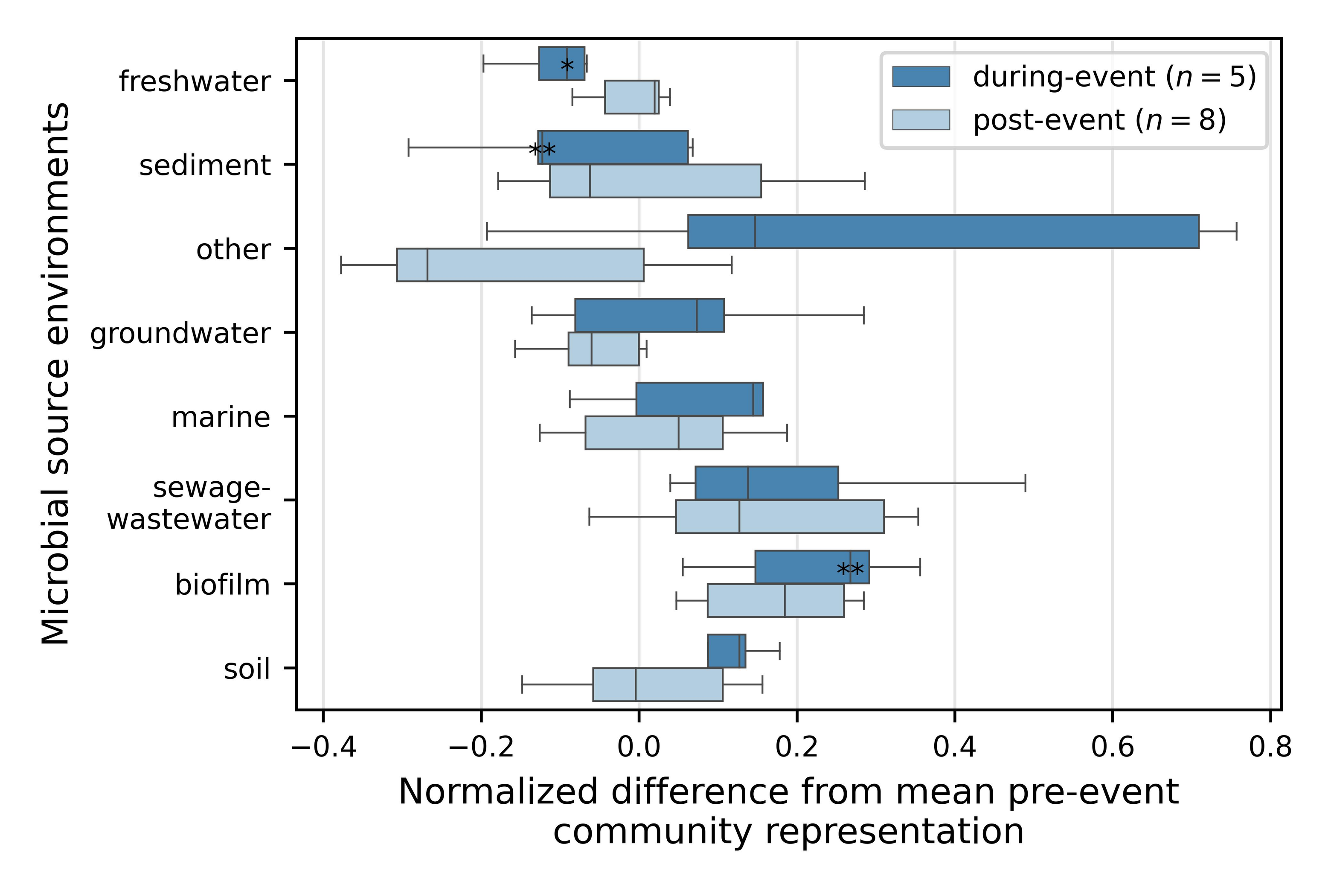

Supplement: S10 Fig — Boxes and whiskers show the distribution of differences in representation from pre-event mean of each source environment in the sampled community during the early and post-event periods. A value of zero indicates no difference from the pre-event community. * p < 0.1, ** p < 0.05 (Mann-Whitney non-parametric U test). (TIF) [file pone.0306896.s012.tif]
